# Skipped *BSCL2* Transcript in Celia’s Encephalopathy (PELD): New Insights on Fatty Acids Involvement, Senescence and Adipogenesis

**DOI:** 10.1371/journal.pone.0158874

**Published:** 2016-07-08

**Authors:** Sofía Sánchez-Iglesias, Alexander Unruh-Pinheiro, Cristina Guillín-Amarelle, Blanca González-Méndez, Alejandro Ruiz-Riquelme, Blanca Leticia Rodríguez-Cañete, Silvia Rodríguez-García, Encarnación Guillén-Navarro, Rosario Domingo-Jiménez, David Araújo-Vilar

**Affiliations:** 1 Thyroid and Metabolic Diseases Unit (U.E.T.eM.), Department of Medicine, Center for Research in Molecular Medicine and Chronic Diseases (CIMUS)-IDIS, University of Santiago de Compostela, Santiago de Compostela, Spain; 2 Unit of Medical Genetics and Dysmorphology, Division of Pediatrics, Hospital Clínico Universitario Virgen de la Arrixaca, Murcia, Spain; 3 Section of Neuropediatrics, Division of Pediatrics, Hospital Clínico Universitario Virgen de la Arrixaca, Murcia, Spain; University of Santiago de Compostela School of Medicine—CIMUS, SPAIN

## Abstract

**Objective:**

PELD (Progressive Encephalopathy with or without Lipodystrophy or Celia’s Encephalopathy) is a fatal and rare neurodegenerative syndrome associated with the *BSCL2* mutation c.985C>T, that results in an aberrant transcript without the exon 7 (Celia seipin). The aim of this study was to evaluate both the process of cellular senescence and the effect of unsaturated fatty acids on preadipocytes from a homozygous c.985C>T patient. Also, the role of aberrant seipin isoform on adipogenesis was studied in adipose-derived human mesenchymal stem cells.

**Material and methods:**

Cellular senescence was evaluated using β-galactosidase staining of preadipocytes obtained from a homozygous c.985C>T patient. Moreover, these cells were cultured during 24 hours with Intralipid, a soybean oil-based commercial lipid emulsion. The expression of the different *BSCL2* transcripts was measured by qPCR. Adipose-derived human mesenchymal stem cells were differentiated to a fat lineage using StemPRO adipogenesis kit, and the expression of *BSCL2* transcripts and several adipogenesis-related genes was measured by qPCR.

**Results:**

the treatment of preadipocytes with unsaturated fatty acids significantly reduced the expression of the *BSCL2* transcript without exon 7 by 34 to 63%. On the other hand, at least in preadipocytes, this mutation does not disturb cellular senescence rate. Finally, during adipocyte differentiation of adipose-derived human mesenchymal stem cells, the expression of adipogenic genes (*PPARG*, *LPIN1*, and *LPL*) increased significantly over 14 days, and noteworthy is that the *BSCL2* transcript without exon 7 was differentially expressed by 332 to 723% when compared to day 0, suggesting an underlying role in adipogenesis.

**Conclusions:**

our results suggest that Celia seipin is probably playing an underestimated role in adipocyte maturation, but not in senescence, and its expression can be modified by exogenous factors as fatty acids.

## Introduction

Seipin is a protein whose function has not been yet fully elucidated. Mutations in *BSCL2* gene are related to type 2 Berardinelli-Seip congenital lipodystrophy (type 2 CGL) [1] and also with various congenital neuropathies [2]. Seipin is a membrane protein of the endoplasmic reticulum (ER). Predictive algorithms and experimental data suggest that it consists of two transmembrane domains, a highly conserved intraluminal loop, and two N-terminal and C-terminal domains in cytoplasm [3].

Under natural conditions, *BSCL2* gene encodes mainly three transcripts of 462 (*BSCL2-003*, ENST00000360796; CCDS44627), 398 (*BSCL2-004/005/006*, ENST00000421906; ENST00000407022; ENST00000403550; CCDS8031) and 287 (*BSCL2-008*, ENST00000278893; CCDS55769) amino acids respectively. The first transcript described [1] was *BSCL2-004/005/006*. *BSCL2-003* is identical to the first one except for a N-terminal extension of 64 amino acids encoded by exon 1. The 287-amino acid short transcript features skipping of exon 7, which results in a change in the reading frame, so that the resulting protein is completely different from the other two ones in the amino acid stretch encoded from exon 6 to exon 10.

The function of seipin is still being investigated, however, its relationship with adipogenesis, with the genesis of lipid droplets and the regulation of the metabolism of phospholipids and triacylglycerides has been established [4–10]. It was also suggested that seipin might play an important role in the nervous system [11–15].

Type 2 CGL syndrome is a rare autosomal recessive disease characterized by an almost total lack of adipose tissue in the body. In addition to the lack of adipose tissue, other clinical features are insulin resistance, liver steatosis and hypertriglyceridemia, which can evolve into diabetes mellitus and early cirrhosis. Type 2 CGL is also characterized by a cognitive delay of variable severity [16]. Moreover, *BSCL2*-associated neuropathies are autosomal dominant disorders affecting upper and/or low motor neuron without affecting life expectancy.

We have previously reported a new seipin-associated neurodegenerative syndrome (PELD, Progressive Encephalopathy with/without Lipodystrophy or Celia’s Encephalopathy, MIM:#615924) [17] due to a c.985C>T mutation in *BSCL2* gene. This mutation gives rise to an alternative splicing leading to skipping of exon 7 and a reading frame shift resulting in a new aberrant protein, hereafter called Celia seipin [17], similar to *BSCL2-008* transcript. Patients suffering PELD die prematurely before age 9 as a consequence of a severe and progressive encephalopathy. Noteworthy, homozygous cases hardly show lipodystrophic features while compound heterozygous cases have a mixed phenotype, neurodegenerative and lipodystrophic.

Despite its recessive pattern of inheritance, we have proposed a toxic gain of function mechanism, so that Celia seipin accumulation in the ER of neurons might induce the unfolded protein response (UPR) responsible for cellular damage and apoptosis [17, 18].

A relevant fact in the natural history of this neurodegenerative condition is that neurological clinical signs do not appear immediately. Thus, affected children are born healthy from a neurological point of view. The homozygous patients manifest psychomotor delay between 12 and 24 months of age and neurological involution becomes evident after 3 years. In compound heterozygotes, lipodystrophy was evident from the first month of life, while the neurological natural history was similar to the homozygous cases. All patients died between 7–8 years, except the youngest one, who is still, at 6.8 years of age, in the early stages of the neurological condition. Given this striking disease evolution, we wondered whether c.985C>T mutation could interfere in the process of cellular senescence. In this line, *in vitro* studies with yeast, have shown the importance of a correct lipolysis for the progression of the cell cycle [19], and mutations in *FLD1*, the orthologous gene of *BSCL2*, decreases lipolysis [10], suggesting a possible molecular relationship with cellular senescence.

On the other hand, all these patients, both homozygous and compound heterozygous, presented, at least during the first months of life, very high levels of plasma triglycerides. It is well known [20] that the contribution of essential fatty acids is key for a normal development of the nervous system in humans, mainly up to 3 years of age, and, as already mentioned, seipin plays a crucial role in the formation of lipid droplets and in the synthesis of phospholipids [4, 5, 7, 21]. Therefore, another hypothesis of this study is that the contribution of fatty acids in the diet might influence the disease evolution of this neurodegenerative process.

In addition, previous studies of our group suggest that *BSCL2-008* transcript might play a positive role in the maintenance of adipogenesis, considering that clinical features of type 2 CGL revert or are not present in homozygous subjects for the c.985C>T mutation [17]. Since no relation between short human *BSCL2* transcript and adipogenesis has been established before, this led us to study the involvement of *BSCL2* transcripts during the differentiation of adipose-derived human mesenchymal stem cells (ADSCs).

## Materials and Methods

This study was approved by the Ethics Review Panel of Xunta de Galicia, and carried out according to the ethical guidelines of the Helsinki Declaration. Written informed consent was obtained from the parents of the participants included in the study.

### Adipose tissue biopsies and cell culture

A small sample of subcutaneous adipose tissue of the abdominal area was obtained from the index case (homozygous for c.985C>T mutation), at 6 years of age. A control adipose tissue sample was obtained, in accordance with ethical regulations of current Spanish legislation, from a 6-year-old control boy who underwent programmed abdominal surgery for cryptorchidism. The collected explants were processed immediately following sampling. Small pieces of adipose tissue were rinsed with PBS and placed on a 60 mm dish (BD Falcon^TM^; Mississauga, ON, Canada) containing Dulbecco’s modified Eagle’s medium (DMEM; Sigma-Aldrich, St. Louis, MO, USA) plus 30% fetal bovine serum (FBS) and gentamicin 50 μg/mL (Sigma-Aldrich), and incubated at 37°C with 5% CO_2_ in a Water-Jacket CO_2_ incubator (NuAire, Plymouth, MN, USA). After one week, preadipocytes that had migrated out of the explants were recognized by the presence of small lipid droplets within the fibroblast-like cells. The explants tissues were then removed from the dishes and the cells were further cultured with fresh medium until a 70% confluent monolayer was obtained. Afterwards, these preadipocytes were tripsinized (TrypLE™ Express Stable Trypsin-like Enzyme with Phenol Red; Gibco Life Technologies; Carlsbad, CA, USA) and cultured on 100 mm dishes in DMEM containing 10% FBS and penicillin-streptomycin 1% (Sigma-Aldrich). Commercially available ADSCs were purchased from Invitrogen (StemPro human adipose-derived stem cells, Invitrogen, Carlsbad, CA, USA). Invitrogen isolated these ADSCs from the abdominal region of a 39-year-old female donor using lipoaspirate, the cells were then expanded for one passage prior to cryopreservation and assayed for purity using flow cytometric analysis of cell surface antigen expression. ADSCs were maintained in tissue culture grade uncoated Petri dishes (BD Falcon^TM^) in complete MesenPRO RS™ medium (Invitrogen) supplemented with 1x GlutaMAX™-I (Gibco) and gentamicin 50 μg/mL, and incubated at 37°C with 5% CO_2_ in a Water-Jacket CO_2_ incubator (NuAire). Culture medium was changed every three days, and the cells were split at 80% confluence. ADSCs were used at low-passage 3 for all experiments.

### Treatment with fatty acids

Preadipocytes were cultured on 35 mm multiwell dishes (6-well plates) in DMEM containing 10% FBS and penicillin-streptomycin 1% supplemented during 24 hours with 83.3 mg/dL Intralipid (200 mg/mL, Fresenius Kabi, Uppsala, Sweden), a soybean oil-based commercial lipid emulsion that is enriched predominantly with polyunsaturated fatty acids (PUFA: 61%); specifically, the fatty acid composition of Intralipid is 54.7% linoleic, 20.9% oleic, 11% palmitic, 6.6% alpha-linolenic and 3.9% stearic [22]. The dose was chosen in agreement to that used in human parenteral nutrition assuming a distribution volume of 5 L.

### Senescence associated β-galactosidase staining

Expression of pH-dependent senescence associated β-galactosidase (SA-β-gal) activity was analyzed simultaneously in different passages of control and index case preadipocytes using the SA-β-gal staining kit (Cell Signaling Technology, Boston, MA) [23]. Cells were cultured in 24-well plates (6.5 mm multiwell dishes) during 24 hours, washed in PBS, fixed for 15 minutes at room temperature in 2% formaldehyde/0.2% glutaraldehyde, washed again, and incubated overnight at 37°C (without CO_2_) with fresh staining solution: 1 mg/mL of 5-bromo-4-chloro-3-indolyl β-D-galactoside (X-Gal) in dimethylformamide, 40 mM citric acid/sodium phosphate, pH 6.0, 5 mM potassium ferrocyanide, 5 mM potassium ferricyanide, 150 mM NaCl, 2 mM MgCl_2_. Cell nuclei were stained with 5 μl/well of 50 μM fluorochrome bisbenzamide (Hoechst 33258; Santa Cruz Biotechnology, Santa Cruz, CA, USA) during 40 minutes at room temperature. Cells were visualized with an Olympus IX51 microscope (Olympus Corporation, Tokyo, Japan) by bright field microscopy and photographed with an Olympus DP72 digital camera using the cellSens software (Olympus Corporation). Quantification was performed with ImageJ software (National Institute of Mental Health) in triplicate, taking 4 random non-overlapping fields of view for each sample.

### Adipogenic differentiation and Oil Red O staining

To induce ADSCs adipogenic differentiation, culture medium was switched to a commercially available differentiation media kit, StemPRO adipogenesis kit (Invitrogen). Culture medium was changed every three days. At day 14 after induction of adipogenic differentiation, ADSCs were stained with Oil Red O (Sigma-Aldrich) to reveal fully differentiated cells. Cells were washed with PBS and fixed in 10% formalin in isotonic buffer for 1 hour at room temperature. After rinsing three times with double distilled H_2_O to remove residual formalin, lipid vacuoles were stained with 0.6% Oil Red O (weight/volume) in 60% isopropanol for 1 hour at 22°C and washed three times with double distilled H_2_O. Following Oil Red O staining, cells were finally visualized with an Olympus IX51 microscope (Olympus Corporation) and photographed with an Olympus DP72 digital camera using the software cellSens (Olympus Corporation).

### RNA extraction and retrotranscription

Total RNA was extracted from preadipocytes and ADSCs using Trizol (Invitrogen) as per the manufacturer’s instructions. RNA was reverse-transcribed using M-MLV reverse transcriptase (Invitrogen) as previously described [24].

### cDNA PCR

*BSCL2* cDNA was amplified with primers designed with the Primer3Plus software (http://www.bioinformatics.nl/cgi-bin/primer3plus/primer3plus.cgi) (forward: 5’-CAGATGCTGGACACACTGGT-3’, reverse: 5’-ATCACTGGCCTCAGGCTCTA-3’). PCR conditions were as follows: 5 min at 95°C followed by 40 cycles, each consisting of 30 s at 95°C, 30 s at 59°C, and 1 min at 72°C, then a 10 min final elongation at 72°C. Amplification fragments obtained were separated out by agarose gel electrophoresis (1%).

### Real-time RT-PCR

Specific primers and probes designed by Universal ProbeLibrary (Roche Diagnostics, Sant Cugat del Valles, Spain) were used in a Light Cycler 2.0 (Roche Diagnostics) to determine specifically expression of adipogenic genes *PPARG*, *LPIN1* and *LPL*, and two distinct *BSCL2* transcripts on days 0, 1, 2, 3, 4, 5, 6, 7, 8 and 14 of adipogenic differentiation. The probe #73 (TCCTCAGC) used with forward: 5’-TGCGCCTTCATAGGTGTTG-3’, and reverse primer: 5’-ACCCACTGCATGTAGCTGAA-3’, hybridizes with exon 7 of *BSCL2* gen (amplicon 74 nucleotides), while the reverse primer: 5’-AGCGATCATTGAGATCCACA-3’, used with the probe #42 (CATCCAGC) and forward primer: 5’-TTTTCGGATGTTAACCTGAGC-3’, exclusively hybridizes with the union of exon 6 and exon 8 (amplicon 96 nucleotides). Real-time RT-PCR conditions are available upon request. Results were normalized for the *RNA polymerase II* or *18s* rRNA genes using the 2-ΔΔ CT method [25].

### Statistical Analysis

The statistical significance was determined using non-parametric Kruskal-Wallis test, followed by a Mann-Whitney U post-hoc with Bonferroni correction. Senescence assay was performed in triplicate, taking photographs of 4 random fields per well, and real-time RT-PCR analyses in duplicate, n = 4. Data are presented as mean ± SD with statistical significance set at P<0.05. All statistical analyses were performed using SPSS for PC (release 22.0; SPSS, Chicago, IL, USA).

## Results

### Exon 7 skipping confirmation

**Fig 1:**
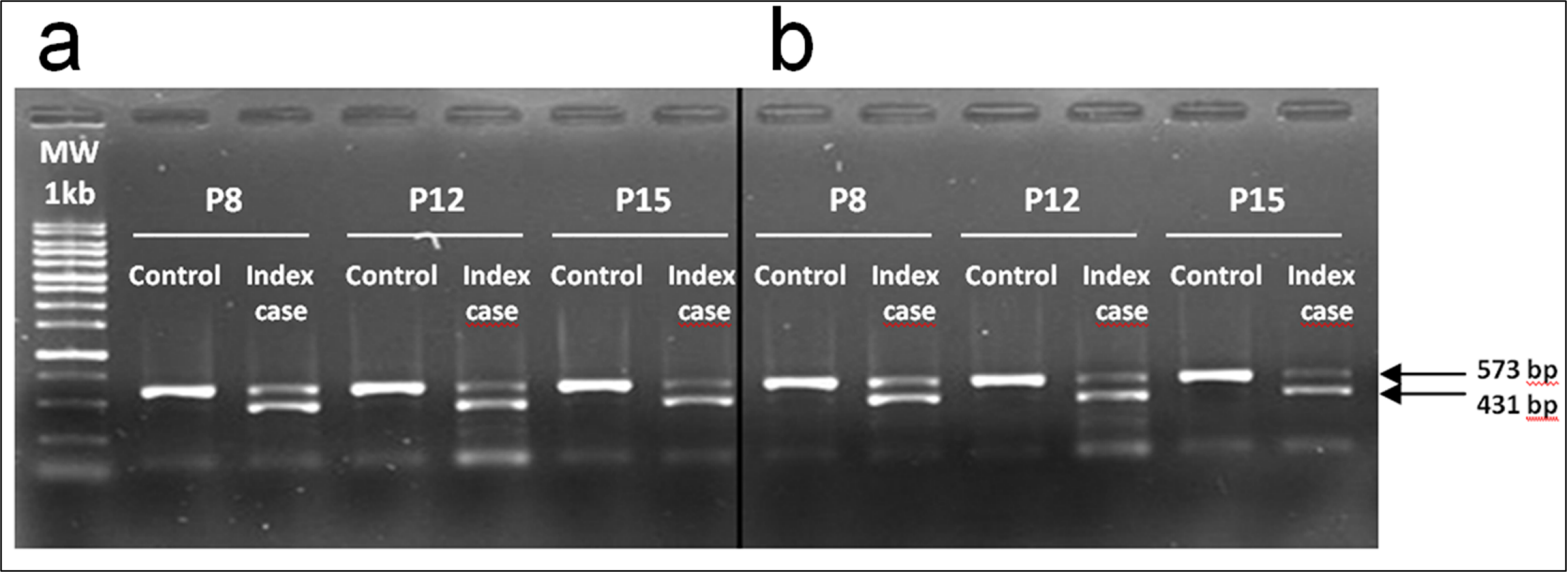
Amplification results of the splicing region. Results of the amplification of the 573 bp region of cDNA from preadipocytes from the index case and control subject in different passages without Intralipid treatment (a) or in the presence of Intralipid (b). Only samples from the index case show the presence of the 431 base pair-long additional band which once sequenced confirmed the skipping of exon 7.

Preadipocytes from index case and control were cultured and analyzed by PCR to study the expression of seipin. As shown in Fig 1A, control cells showed a single band in electrophoresis, while the index case samples showed a second band of lower molecular weight, corresponding to the exon 7 skipped Celia transcript, in agreement with our previous study [17]. Moreover, this electrophoretic differential pattern remained identical at all passages. Similarly, after treatment with Intralipid, the electrophoretic pattern did not change in the different passages of both control and index case samples (Fig 1B).

### Expression of seipin transcripts

The basal expression of the transcripts containing exon 7 was much higher (72-fold, passages average) than that of the exon 7-skipped transcript in control cells, while this was not so in cells from the index case, in which the expression of the two transcripts was in a similar order (Table 1A). At basal conditions and as expected, the relative expression of the large transcripts was reduced in preadipocytes with the c.985C>T *BSCL2* Celia mutation (13%) as compared with control cells, however, the expression of the skipped transcript in mutant cells was around 10-17-fold higher than that was observed in control cells (Table 1A).

**Table 1:** Relative expression of *BSCL2* transcripts.

**A) Relative expression of BSCL2 transcripts at basal conditions**				
	**CONTROL**		**INDEX CASE**	
	With exon 7	Without exon 7	With exon 7	Without exon 7
**P8**	1.034±0.275	0.012±0.004 *	0.136±0.035 ^**§**^	0.120±0.026 ^**†**^
**P12**	1.036±0.302	0.017±0.005 *	0.164±0.028 ^**§**^	0.282±0.062 ^**†**^ ^**‡**^ ^**#**^
**P15**	1.109±0.321	0.016±0.006 *	0.172±0.051 ^**§**^	0.195±0.059 ^**†**^
**B) Relative expression of BSCL2 transcripts after Intralipid treatment**				
	**CONTROL**		**INDEX CASE**	
	With exon 7	Without exon 7	With exon 7	Without exon 7
**P8**	1.096±0.214	0.013±0.002 *	0.101±0.017 ^**§**^	0.079±0.011 ^**†**^
**P12**	1.412±0.291 ^**‡**^	0.012±0.002 *	0.142±0.030 ^**§**^	0.102±0.029 ^**†**^
**P15**	1.286±0.236	0.010±0.002 *	0.205±0.036 ^**§**^ ^**‡**^	0.103±0.020 ^**†**^ ^**‡**^ ^**#**^

Relative expression of *BSCL2* transcripts referred to *BSCL2* transcripts with exon 7 in control (A) at basal conditions, and (B) after Intralipid treatment: skipped transcript referred to *BSCL2* transcripts containing the exon 7 in control

*: P<0.05; skipped transcript referred to *BSCL2* transcripts containing the exon 7 in index case

#: P<0.05; transcripts with exon 7 in index case referred to *BSCL2* transcripts containing the exon 7 in control

§: P<0.05; skipped transcript in index case referred to *BSCL2* skipped transcript in control

†: P<0.05

‡: P<0.05 vs. P8. P: passage. All samples were analyzed in duplicate, n = 4. Results were normalized for the *RNA polymerase II* gene.

**Fig 2:**
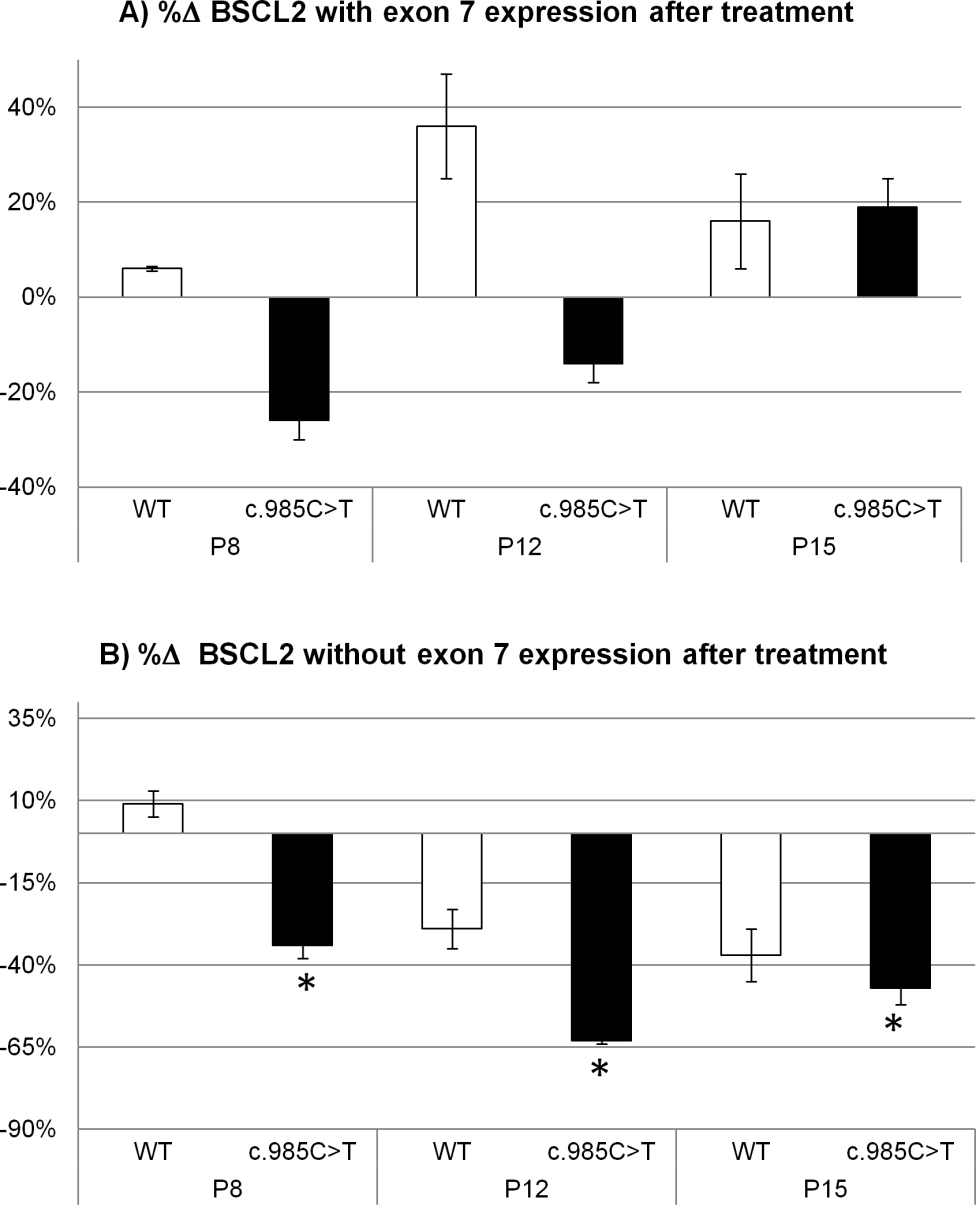
Relative expression and percentage of change in expression of *BSCL2* transcripts after Intralipid treatment. (a) Percentage of change referred to the relative expression of *BSCL2* transcripts with exon 7 and (b) without exon 7, in WT and index case preadipocytes, normalized to the *RNA polymerase II* gene. White bar: WT; black bar: index case. All samples were analyzed in duplicate, n = 4, *:P<0.05.

Regarding real time RT-PCR studies of control and index case preadipocytes, no significant differences were detected in the relative expression of the transcripts containing exon 7 after Intralipid treatment nor in the control neither in the index case cells (Fig 2A, Table 1B), while there were significant differences in the relative expression of the skipped transcript in the index case preadipocytes (Fig 2B, Table 1B). Thus, an unsaturated fatty acid enriched medium significantly reduced (-48% on average for the different passages) the expression of the transcript without exon 7.

### Senescence studies

**Fig 3:**
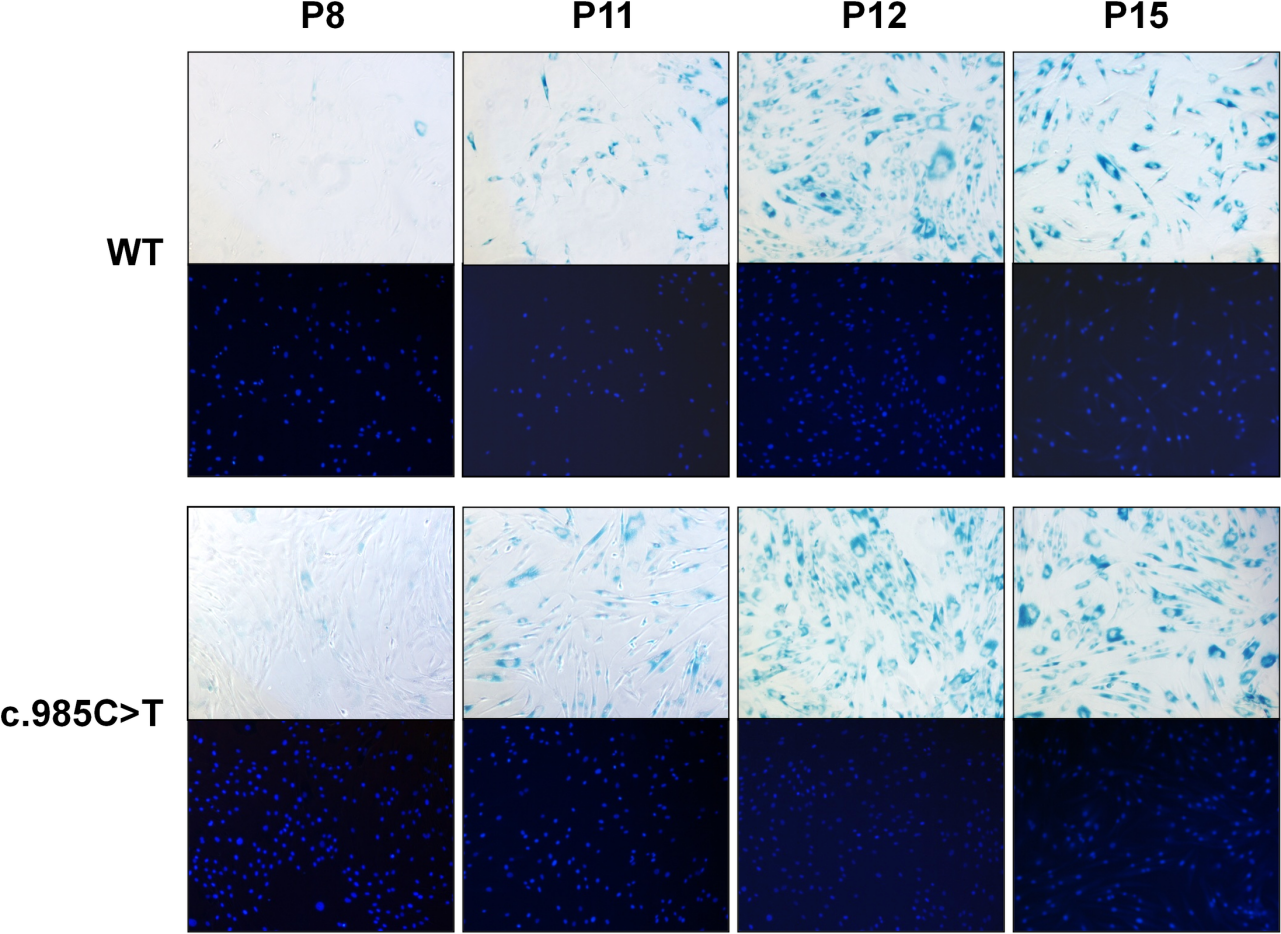
Representative photographs of SA-β-gal activity in preadipocytes. Early and late passages preadipocytes of control (WT) and index case (c.985C>T) were subjected to *in situ* SA-β-gal staining at pH 6 and examined by bright field microscopy. The nuclei were counterstained with Hoechst 33258. Senescent and total cells were quantified in triplicate with ImageJ software, taking 4 random non-overlapping fields of view for each sample. Original magnification ×10.

The percentage of senescent cells was calculated throughout the different passages in order to assess a possible relationship of seipin and the c.985C>T mutation with cellular senescence. Senescent and total cells were counted using the β-galactosidase and Hoechst 33258 dyes, respectively (Fig 3). Little SA-β-galactosidase activity was detected in control and index case cells at P8 (P: passage, 3.9% and 3.4%, respectively), but the number of senescent cells increased continuously and significantly through serial passages both in control and index case cells as evidenced by increased levels of senescence-associated β-galactosidase activity (Fig 3), which increased approximately 20-to 25-fold, accounting for 76–98% of all index case cells, and 23-to 29-fold, accounting for 78–99% of all control cells. This result was expected as continuous cell division is known to promote replicative senescence of cells in vitro [26]. No significant differences between index case and control preadipocytes were seen when cells at the same passage were compared (Fig 3).

### Expression of adipogenic genes after ADSCs differentiation

**Fig 4:**
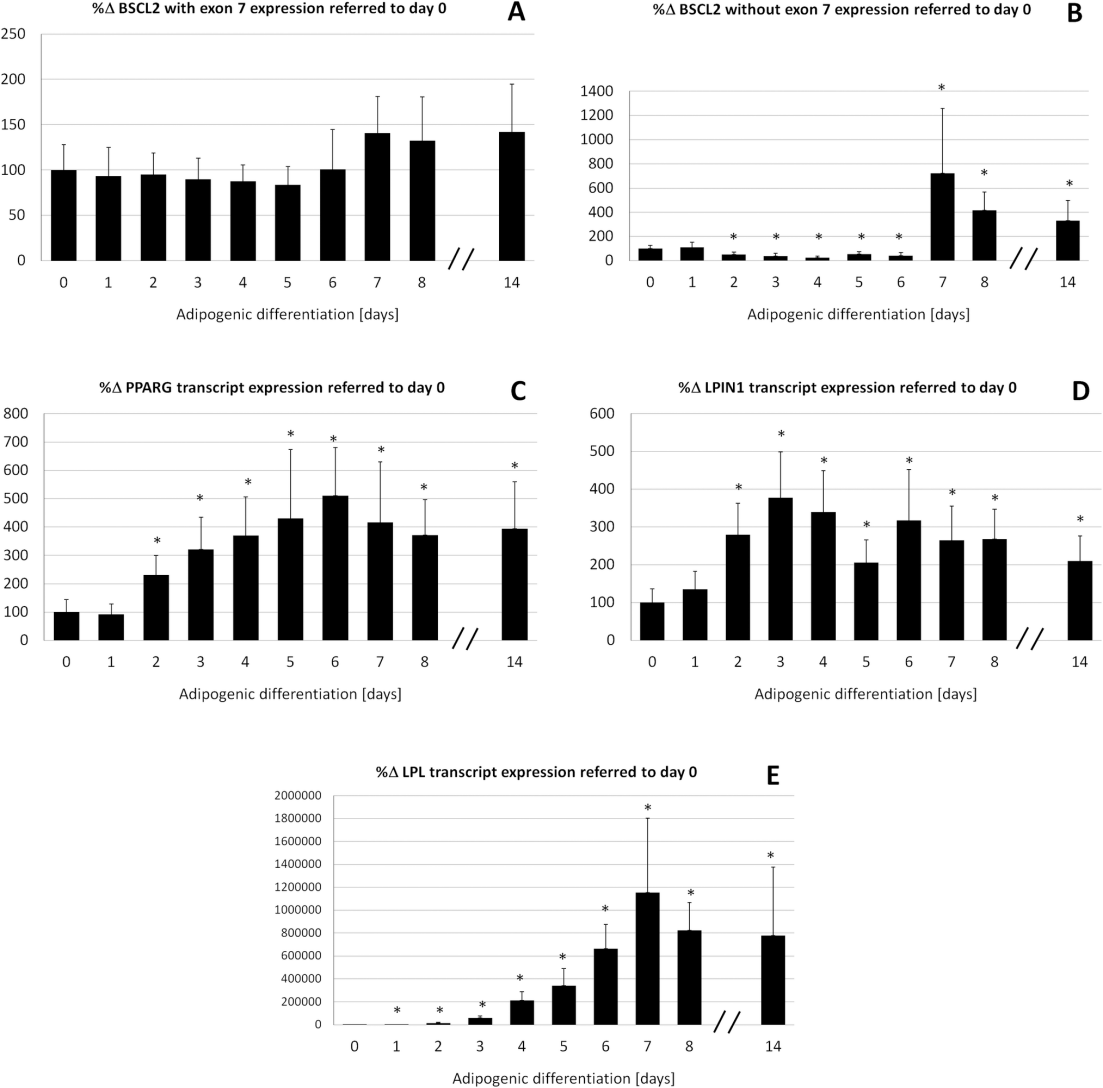
Percentage of change in expression of *BSCL2* transcripts and adipogenic genes. Percentage of change in expression of *BSCL2* transcripts and adipogenic genes referred to day 0, (a) *BSCL2* with exon 7 (b) *BSCL2* without exon 7 (c) *PPARG*, (d) *LPIN1*, (e) *LPL*, in ADSCs (differentiated over 14 days into adipocytes) normalized to the *18S* gene. All samples were analyzed in duplicate, n = 4, *:P<0.05.

Differentiation of ADSCs into adipocytes over 14 days led to accumulation of lipid droplets that were stained positively by Oil red O (S1 Fig). Real time RT-PCR studies were used to determinate the expression of *BSCL2* transcripts and adipogenic genes (Fig 4). As expected, ADSCs differentiation toward the adipogenic lineage induced significant increase in the expression of genes related to adipogenesis: when referred to day 0, *PPARG* and *LPIN1* increased approximately 2-to 5-fold and 2-to 3-fold, respectively, while the expression of *LPL*, an early marker of adipogenic differentiation, was much higher and increased 7-to 11500-fold (Fig 4C–4E). The expression of the *BSCL2* transcripts containing exon 7 did not change significantly over differentiation, while large significant changes were observed with the *BSCL2* skipped transcript whose expression increased notably from 3-to 7-fold since day 7 when compared to day 0 (Fig 4A and 4B).

## Discussion

To date, only 6 patients with Celia’s Encephalopathy have been reported [17] representing a unique and intriguing disease. Homozygous patients did not show lipodystrophy, whereas compound heterozygous subjects had a mixed phenotype, lipodystrophic and neurological [17]. On the other hand, homozygous patient for c.985C>T mutation showed hypertriglyceridemia and hepatic steatosis during their first months of life which were easily corrected with a animal fat-free diet in few months. Although all of these patients suffered an early psycho-motor delay, it was not until 3–4 years of age when these children started their neurological involution leading them to die in the first decade of life.

Although the most parsimonious explanation for the neurological PELD natural history is a progressive loss of neurons affecting first the most evolved brain areas, a putative role for environmental factors should not be ruled out. On the other hand, there is no information about the role of senescence in the pathogenesis of this disease.

It is well known that essential fatty acids have an important influence in normal brain development. So, unsaturated fatty acids, are important in neurogenesis as they have been implicated as critical nutritional factors for appropriate neural function and development [27–30]. Indeed, it was shown that a huge development takes place in the infant brain: an extensive growth including vast axonal and dendritic arborization. Synapses are rich in ω-3 docosahexaenoic acid (DHA), which is proven to be essential in dendritic complexity, neurite outgrowth and neurotransmitter metabolism [30].

985C>T *BSCL2* mutation gives rise to an alternative splicing site, and in this study, treatment with Intralipid significantly reduced its expression by half. Although, for obvious reasons, our experiments were not conducted in neurons, these results may suggest a possible therapeutical approach using a high PUFA percentage in the diet of these patients, assuming a similar behavior of neural cells. Other studies support the possibility that fatty acids can alter splicing events: so, ceramides, a class of long-chain lipid fatty acids, were shown to modify the alternative splicing in different genes [31–33]; DHA, the most abundant ω-3 long-chain PUFA in nerve cells, is particularly effective at regulating *GPX4* splicing variants [34] and the liver fatty acid desaturase alternative transcript expression [35, 36]. On the other hand, a new role for seipin in brain has been proposed, and this protein seems to be involved in the mobilization of lipids to the developing brain [15]. Lastly, studies in mice have demonstrated that dietary deficit of PUFA results in alterations of nerve impulse conduction in retina, learning, and function of the sodium potassium pump [37], accompanied by a DHA decline in membranes [38]. Furthermore, supplementation with ω-3 fatty acids improves memory and learning in rats [39].

It is important to underline that seipin is a key protein in lipid droplet formation [7, 9, 40], but no studies have deepened in the role of *BSCL2* transcripts in this process. On the other hand, others [11, 40, 41] have reported that this protein can have tissue-specific functions. In this study, we found that during adipogenesis of adipose-derived human mesenchymal human stem cells the skipped transcript was the most differentially expressed (>700%) compared to the large *BSCL2* transcripts, suggesting a putative and unknown role in adipogenesis. These results apparently differ from those reported by Payne et al. [8], who found that *BSCL2* expression increased during adipogenesis in human preadipocytes since the first day; however the lack of information about both the transcript they measured and the statistical analysis performed makes difficult to interpret this apparent discrepancy with our results.

Considering the minor clinical features of generalized lipodystrophy present in homozygous subjects for the c.985C>T mutation, it seems that Celia seipin might play some role in adipocyte differentiation, besides being potentially harmful to a correct neuron function, being environmental factors as PUFA important actors in these processes.

Finally, our results established that the mechanism of cellular senescence induction does not alter the seipin transcripts expression and neither mutant cells exhibit a higher senescence rate, suggesting that cellular senescence does not appear to be associated to the pathogenesis of this new disease, on the contrary to what happens with apoptosis [42]. This agrees with the finding that inhibitors of the ER stress significantly inhibit apoptosis, but have no effect in cell senescence [43].

In summary, our study provide strong evidence that *in vitro* treatment with unsaturated fatty acids reduces the expression of the Celia seipin in primary cultures of preadipocytes with c.985C>T mutation in *BSCL2* and that this mutation does not alter the rate of senescence of these cells. We are fully aware that our paper presents a limitation that should be taken into account. The cell model chosen, preadipocytes, does not turn out to be the most suitable to study a process that primarily affects the central nervous system, but the difficulties to obtain mutated human neuronal cultures are obvious. Taking into account this limitation, the results suggest that Celia seipin might be playing a fundamental and to date unknown role in adipogenesis. However, the underlying molecular process whereby this intriguing transcript exert its function remains to be elucidated as does its pathogenic mechanism.

## Supporting Information

S1 Fig Representative photographs of lipid droplets formation in ADSCs demonstrated using Oil Red O staining and light microscopy. (PDF)
