# Supplementary material for: Skipped BSCL2 Transcript in Celia’s Encephalopathy (PELD): New Insights on Fatty Acids Involvement, Senescence and Adipogenesis
Source: PLoS One. 2016 Jul 8;11(7):e0158874. doi: 10.1371/journal.pone.0158874 (PMC4938205; doi:10.1371/journal.pone.0158874)
Supplement: S1 Fig — (PDF) [file pone.0158874.s001.pdf]

Supporting Information

Supplementary Figure 1

Representative photographs of lipid droplets formation in ADSCs demonstrated using Oil Red O staining and light microscopy.

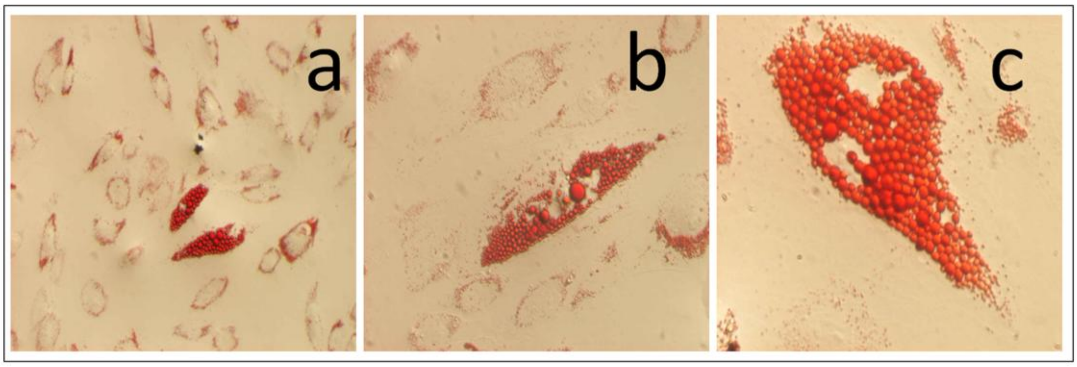

ADSCs were differentiated over 14 days into adipocytes with the commercially available differentiation media kit, StemPRO adipogenesis kit (Invitrogen). At day 14 after the induction of adipogenic differentiation, microphotographs were taken from a central area of a representative triplicate well. Small lipid droplets stained with Oil Red O, located in the cytoplasm of the cells during early incubation, became large due to lipid accumulation. Original magnifications: (a) ×10, (b) ×20, (c) ×40 (magnification of the oculars: ×10).
